# The influence of the municipal human development index and maternal education on infant mortality: an investigation in a retrospective cohort study in the extreme south of Brazil

**DOI:** 10.1186/s12889-021-10226-9

**Published:** 2021-01-22

**Authors:** Carolina Ribeiro Anele, Vânia Naomi Hirakata, Marcelo Zubaran Goldani, Clécio Homrich da Silva

**Affiliations:** 1 Programa de Pós Graduação em Saúde da Criança e do Adolescente - Faculdade de Medicina, Universidade Federal do Rio Grande do Sul (UFRGS), Rua Ramiro Barcelos, 2400, Porto Alegre, RS 90035003 Brazil; 2 Grupo de Pesquisa e Pós-Graduação (GPPG) do Hospital de Clínicas de Porto Alegre (HCPA), Rua Ramiro Barcelos, 2400, Porto Alegre, RS 90035003 Brazil; 3 Serviço de Pediatria e de Atenção Primária em Saúde, Hospital de Clínicas de Porto Alegre (HCPA), Rua Ramiro Barcelos, 2350, Porto Alegre, RS 90035007 Brazil; 4 Departamento de Pediatria - Faculdade de Medicina, Universidade Federal do Rio Grande do Sul (UFRGS), Rua Ramiro Barcelos, 2400, Porto Alegre, 90035003 RS Brazil

**Keywords:** Infant mortality, Live birth, Vital statistics, Human development, Education

## Abstract

**Background:**

Infant mortality is considered an important and sensitive health indicator in several countries, especially in underdeveloped and developing countries. Most of the factors influencing infant mortality are interrelated and are the result of social issues. Therefore, this study performed an investigation of the influence of the MHDI and maternal education on infant mortality in a capital in the extreme south of Brazil.

**Methods:**

It is a retrospective cohort study with data on births and deaths in the first year of life for the period of 2000–2017. The association between the independent variables and the outcome was done by bivariate analysis through simple Poisson regression. The variables that can potentially be considered confounding factors were used in a multiple Poisson regression for robust variances - adjusted model.

**Results:**

The study included 317,545 children, of whom 3107 died. The medium MHDI showed associated with infant death in the first year of life. Maternal education, individually and jointly analyzed with the MHDI, showed association with the outcome of infant death in the first year of life, particularly for children of mothers with lower maternal education (*p* < 0.001). In relation to other related factors, maternal age; number of Prenatal Care Consultations; gestational age, weight, gender and Apgar Index (5th minute) of the newborn showed association with IM (*p* < 0.001).

**Conclusions:**

The HDI is considered a good predictor of infant mortality by some authors and the analyzes of the present study also confirm an association of the medium MHDI and its low MHDIE component with infant mortality. In addition, it was maternal education with less than 8 years of study that that demonstrated a higher risk of death, revealing itself to be a social determinant with a relevant impact on infant mortality. Thus, it is possible to conclude that maternal education is available information, and it is superior to the MHDI to assess the infant mortality outcome.

**Supplementary Information:**

The online version contains supplementary material available at 10.1186/s12889-021-10226-9.

## Background

Infant mortality (IM) is considered, internationally, as one of the most sensitive health indicators, since it is related to social conditions and can also be influenced by prenatal and perinatal factors, as well as environmental factors, especially nutritional and infectious ones [1–3]. Worldwide, countries have dedicated themselves to achieving Millennium Development Goal 4 (MDG 4), which was to reduce by two-thirds the mortality of children under five by the year of 2015. Currently, efforts are focused on the new agenda in which Millennium Development Goal 3 (MDG 3) keeps the reduction of under-five mortality to at least 25 per 1000 live births as a priority [4]. In Brazil there was a significant reduction in IM in the period from 1990 to 2012, when MDG 4 was achieved 3 years before the deadline [5, 6]. However, as in other countries, neonatal deaths still remained high and corresponded to approximately 70% of mortality in the first year of life [1, 7]. Porto Alegre, the state capital located in the extreme south of Brazil and where the present study was carried out, presented a variation in the infant mortality rate from 18.22 in 1994 to 9.13 deaths per thousand live births in 2018, which represented a decrease of 49.89% [8]. For the epidemiological surveillance of such vital events, in Brazil, the Live Births Information System (SINASC) and the Mortality Information System (SIM) are used [9].

Most of the factors influencing IM are interrelated and are the result of social issues, including the educational level of the mother, which is an important social determinant for better child health conditions because it leads to a better maternal perception of health needs and problems. Maternal education seems to be directly related to the healthy growth and development of the child [10, 11]. The region of residence is also related, albeit indirectly, to IM, being an important social factor in child health outcomes [12], as less developed regions may have worse physical structure and sanitation conditions and limited access to health services [3, 13, 14].

There are important differences in these factors between countries, especially in developing countries like Brazil, where there are differences between regions according to socio-economic characteristics, with significant social inequities. As an alternative to assess these development disparities in different countries, the Human Development Index (HDI) evaluates human development through three indicators: schooling (expected years of schooling for children of school age and average schooling in years for adults over 25), longevity (life expectancy at birth) and income (per capita income). In this context, the Municipal Human Development Index (MHDI) was launched in Brazil in 2013. It was a strategy to adapt the HDI to the local context, allowing the calculation of HDI at the municipal level of the metropolitan regions of Brazil. MHDI considers the same three dimensions of Global HDI (longevity, education and income), including its three components, MHDI Income (MHDII), MHDI Longevity (MHDIL), MHDI Education (MHDIE), but it is adapted to the national context and uses local indicators available for the calculation. In the MHDI, the geometric mean is used: the dimensions (longevity, income and education) are multiplied and the product is extracted by the cube root, for this calculation the three components have the same weight [15]. The general MHDI of Porto Alegre was 0.805 in 2010, ranking as the 7th best Brazilian capital [15].

Because it is a sensitive indicator for socioeconomic and environmental changes, IM should be constantly monitored, and a better understanding of the association of HDI and maternal education with IM assists in the planning of actions and policies aimed at maternal and child health, particularly in regions of greater social vulnerability [1, 3]. Therefore, this study performed an investigation of the influence of the MHDI and maternal education on infant mortality in a capital in the extreme south of Brazil.

## Methods

### Study design and participants

This is a retrospective cohort study that used the annual SIM and SINASC databases provided by the Non transmissible Vital Events, Diseases and Aggravates Surveillance Team of the General Health Surveillance Coordination of the Porto Alegre Municipal Health Secretariat.

The city of Porto Alegre has an estimated population of 1,479,101 people and has eight Planning Management Regions that are subdivided into seventeen Participatory Budget Regions (macro-regions), each of which is made up of a set of neighborhoods with affinities to each other [8], it has an area of 471.85km^2^. In relation to education level, in 2017, the illiteracy rate was 2.27%, the Basic Education Development Index (IDEB) was 4.9 (target 5.5) for the initial years and 3.9 (target 4.7) for the final years. Regarding mothers heads of families from 2000 to 2010, the percentage of women without complete primary education showed a decrease of 10%, from 39.87 to 29.15% [8].

The study included all live births in the city of Porto Alegre from January 2000 to December 2016 obtained at SINASC and all deaths of children under 1 year of age from January 2000 to the month December 2017, obtained from SIM. Thus, all children were included in the study until they completed their first year of life. A linkage was made between the two databases through the number of the Declaration of Live Birth (DLB), that refers to document filled in at the maternity hospital when the child is born, the name of the mother and the date of birth, in order to establish a unique database. After a first automatic attempt, there was no agreement on the number of DLB of 269 children who died in the first year of life (7.2% of total deaths) between the two databases. From then on, a manual search was carried out in an attempt to correct and locate the number of the DLB, through the name of the mother, which was confirmed by the date of birth and weight of the newborn and, in a few cases, the address of the maternal residence. From this process, a further 165 deaths were located (4.4% of the total deaths of children in the first year) and the DLB numbers were corrected according to the SINASC database, totaling a final loss of 104 children (2.8% of the total deaths).

After performing the linkage between the SINASC and SIM databases, the newborns twins or higher (2.5% of all live births in the period studied) were excluded to avoid double maternal data and to avoid confusion bias as they are a population that represents greater use of assisted conception, a method used in the private sector. Newborns with birth weight of less than 500 g (0.09% of all live births) also were excluded following publications from other studies investigating infant mortality. Also excluded who were not born in the municipality of Porto Alegre (0.65% of live births) and who had their maternal address ignored (0.36% of live births).

Although there may have been double entry of mothers due to the effect of the long period analyzed, it was considered that, eventually, they may have changed homes and also improved their level of education. However, it is important to note that, throughout the study period, the percentage of mothers who were under 18 years of age and, therefore, with greater possibility of changing their level of education, was only 7,8%. In addition, the study population is based on the newborn that will become a new case even if it is a second child of a given mother.

From SINASC, the following variables were used: number of Declaration of Live Birth; maternal age (10–17, 18–34, ≥35 years); maternal education in completed years of study (< 8; 8–11; ≥12 years); maternal home district (see Additional file 1) [16]; Planning Management Region of Maternal Residence; Participatory budgeting Regions of Maternal Residence, which were later called macro-regions (see Additional file 2 and Additional file 3) [16]; maternal marital status (married/stable union, single/separated/widowed); number of previous living children; number of previous dead children (none; ≥1); number of Prenatal Care Consultations (None; 1–3; 4–6; ≥7); gestational age in weeks (< 27, 28–31, 32–36, ≥37); type of hospital (public, private, mixed); type of delivery (vaginal, cesarean); weight of the newborn (grams); Apgar rate at the 5th minute of life (≥7, < 7); gender of the newborn (female, male). The study considered the maternal data at birth contained in the database of SINASC, since this system presents a greater completeness of the data, mainly in the city of the study that presents itself as one of the capitals with the best coverage and filling of this system [9, 11]. From SIM, the variables used were: the number of the Declaration of Live Birth and date of death. The following variables were also introduced in the bank: year; parity, created from the number of live children and number of dead children (primipara, multipara) and the value of the MHDI of the macro-regions of maternal domicile and its three components: MHDI Income (MHDII), MHDI Longevity (MHDIL) and MHDI Education (MHDIE). The study used the following cutoff points previously calculated and established by the United Nations Development Program Brazil (UNDP Brazil), the Institute for Applied Economic Research (IPEA) and the João Pinheiro Foundation, classified as very low (0–0.499), low (0.5–0.599), medium (0.6–0.699), high (0.7–0.799) and very high (0.8–1.0) and available through the Atlas Human Development online platform at Brazil [15]. The values ​​of each component and the MHDI were calculated based on the 2010 Demographic Census of the Brazilian Institute of Geography and Statistics (IBGE). In the municipality studied, no macro-region has an MHDI lower than 0.6. Thus, according to the cutoff points already established, the MHDI of the macro-regions was classified only as medium, high and very high. The exception was only for the MHDIE component, which had a low rating in some macro-regions of the study.

### Statistical analyses

Preliminarily, a descriptive analysis of variables with absolute and relative frequencies was presented. The association of variables of the study, according to the MHDI classification, with the death outcome was performed by Chi-square test. Later, the association between the determinants involved (independent variables) with the outcome of death was verified by a bivariate analysis through simple Poisson regression. Finally, the variables that can potentially be considered confounding factors, as well as maternal education, were used in four models adjusted to estimate the influence of MHDI, MHDII, MHDIL and MHDIE on infant mortality in a multiple Poisson regression for robust variances.

### Ethical aspects

The study was approved by the Research Ethics Committees of Hospital de Clínicas of Porto Alegre and the Research Ethics Committees of Municipal Health Department of Porto Alegre, respectively, under the respective protocol numbers 2,940,235 and 3,153,671.

## Results

**Fig. 1:**
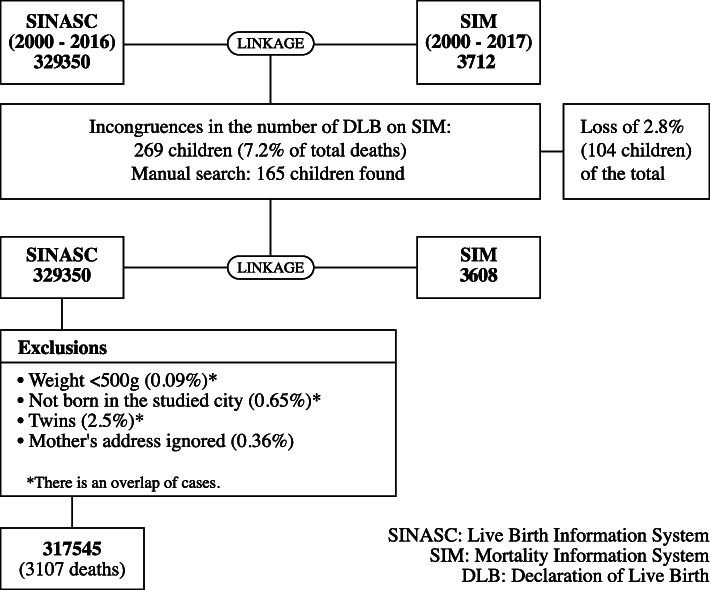
Description of the study population: live births and infant deaths in the first year of life in the municipality of Porto Alegre, Brazil (2000–2017). figure made by the author. *Source: figure made by the author*

Three hundred seventeen thousand five hundred forty-five children were included in the study. Of these 3107 died in the first year of life (Fig. 1).

The proportion of mothers aged 35 years or older increases according to the MHDI classification, being higher (25.2%) in those residing in macro-regions with very high MHDI. Independent of the MHDI classification, a maternal age under 18 years showed association with child death in the first year of life (*p* < 0.05). In macro-regions with high and very high MHDI, single mothers, judicially separated or widowed, multiparas and with one or more previous dead children also showed association with death (p < 0.05). Regardless of the MHDI classification, the lower the number of Prenatal Care Consultations the higher the percentage of deaths. The percentage of cesarean sections was higher in macro-regions with high and very high MHDI (43 and 60.4%, respectively). In the medium and high MHDI classifications, cesarean section showed a significant association with death, while in very high MHDI vaginal delivery showed a significant association with death. In the three classifications of MHDI (medium, high and very high), the proportion of deaths was higher for those newborns with a lower gestational age, with low weight or with an Apgar Index of less than 7 at the fifth minute of life. Independent of the MHDI classification have congenital anomaly showed an association with child death in the first year of life (*p* < 0.05). The public hospital showed a significant association with death, regardless of the MHDI classification (Table 1). In the three classifications of the MHDI, there is a decrease in MI in the city of Porto Alegre during the study period (see Additional file 4).

**Table 1:** Distribution of the absolute frequency of sociodemographic, perinatal and neonatal characteristics with the respective mortality percentages according to the MHDI classification in Porto Alegre (Rio Grande do Sul, Brazil) from 2000 to 2017

	MHDI					
	MEDIUM n(%)		HIGH n(%)		VERY HIGH n(%)	
	TOTAL	DEATH	TOTAL	DEATH	TOTAL	DEATH
**Maternal Age**						
10 to 17 years	5540 (10.6)	86 (1.6)^a^	15,708 (8.6)	218 (1.4)^a^	3628 (4.4)	66 (1.8)^a^
18 to 34 years	40,411 (77.5)	446 (1.1)	140,685 (77.2)	1358 (1.1)	58,530 (70.5)	436 (0.7)
≥ 35 years	6170 (11.9)	80 (1.3)	25,859 (14.2)	280 (1.1)	20,907 (25.2)	132 (0.6)
	**52,121 (100)**	**612 (1.2)**	**182,252 (100)**	**1856 (1.1)**	**83,065 (100)**	**634 (0.8)**
**Maternal Education**						
< 8 years	20,301 (39.2)	301 (1.5)^a^	59,084 (32.5)	867 (1.5)^a^	13,486 (16.3)	205 (1.5)^a^
≥ 8 to 11 years	24,981 (48.2)	251 (1.0)	83,658 (46.1)	752 (0.9)	25,528 (30.8)	216 (0.8)^a^
≥ 12 years	6541 (12.6)	50 (0.8)	38,806 (21.4)	224 (0.6)	43,780 (52.9)	205 (0.5)
	**51,823 (100)**	**602 (1.2)**	**181,548 (100)**	**1843 (1.0)**	**82,794 (100)**	**626 (0.8)**
**Marital Status**						
Single/Separated/Widowed	37,022 (71.3)	452 (1.2)	123,195 (67.8)	1428 (1.2)^a^	43,144 (52.1)	434 (1.0)^a^
Married/stable union	14,937 (28.7)	156 (1.0)	58,639 (32.2)	416 (0.7)	39,739 (47.9)	192 (0.5)
	**51,959 (100)**	**608 (1.2)**	**181,834 (100)**	**1844 (1.0)**	**82,883 (100)**	**626 (0.8)**
**Parity**						
Primipara	20,293 (39)	221 (1.1)	78,595 (43.2)	685 (0.9)	43,217 (52.1)	273 (0.6)
Multipara	31,684 (61)	388 (1.2)	103,341 (56.8)	1162 (1.1)^a^	39,707 (47.9)	355 (0.9)^a^
	**51,977 (100)**	**609 (1.2)**	**181,936 (100)**	**1847 (1.0)**	**82,924 (100)**	**628 (0.8)**
**Number of Dead Children**						
≥ 1	5038 (9.7)	67 (1.3)	17,376 (9.5)	225 (1.3)^a^	8038 (9.7)	83 (1.0)^a^
None	47,067 (90.3)	545 (1.2)	164,833 (90.5)	1630 (1.0)	75,008 (90.3)	550 (0.7)
	**52,105 (100)**	**612 (1.2)**	**182,209 (100)**	**1855 (1.0)**	**83,046 (100)**	**633 (0.8)**
**Number Prenatal Care Consultations**						
None	1844 (3.6)	78 (4.2)^a^	6048 (3.3)	286 (4.7)^a^	1808 (2.2)	92 (5.1)^a^
from 1 to 3	5530 (10.7)	137 (2.5)^a^	15,889 (8.7)	427 (2.7)^a^	4285 (5.2)	103 (2.4)^a^
from 4 to 6	13,463 (26.0)	192 (1.4)^a^	40,524 (22.3)	518 (1.3)^a^	11,887 (14.3)	164 (1.4)^a^
7 and more	30,993 (59.8)	199 (0.6)	119,233 (65.6)	605 (0.5)	64,885 (78.3)	266 (0.4)
	**51,830 (100)**	**606 (1.2)**	**181,694 (100)**	**1836 (1.0)**	**82,865 (100)**	**625 (0.8)**
**Birth**						
Vaginal	33,379 (64.0)	361 (1.1)	103,908 (57.0)	1007 (1.0)	32,869 (39.6)	300 (0.9)^a^
Cesarean	18,744 (36.0)	252 (1.3)^a^	78,340 (43.0)	848 (1.1)^a^	50,196 (60.4)	334 (0.7)
	**52,123 (100)**	**613 (1.2)**	**182,248 (100)**	**1855 (1.1)**	**83,065 (100)**	**634 (0.8)**
**Gestational Age**						
< 27 weeks	232 (0.4)	133 (57.3)^a^	790 (0.4)	465 (58.9)^a^	295 (0.4)	151 (51.2)^a^
28 to 31 weeks	473 (0.9)	85 (18.0)^a^	1523 (0.8)	234 (15.4)^a^	634 (0.8)	78 (12.3)^a^
32 to 36 weeks	4331 (8.3)	123 (2.8)^a^	14,926 (8.2)	349 (2.3)^a^	6979 (8.4)	115 (1.6)^a^
37 or more	46,967 (90.3)	261 (0.6)	164,641 (90.7)	764 (0.5)	75,032 (90.5)	276 (0.4)
	**52,003 (100)**	**602 (1.2)**	**181,880 (100)**	**1812 (1.0)**	**82,940 (100)**	**620 (0.7)**
**Apgar At 5 Minutes**						
< 7	686 (1.3)	168 (24.5)^a^	2205 (1.2)	592 (26.8)^a^	694 (0.8)	179 (25.8)^a^
≥ 7	50,961 (98.7)	410 (0.8)	178,989 (98.8)	1158 (0.6)	82,091 (99.2)	416 (0.5)
	**51,647 (100)**	**578 (1.1)**	**181,194 (100)**	**1750 (1.0)**	**82,785 (100)**	**595 (0.7)**
**Low Weight**						
No (≥2500 g)	47,425 (91.0)	262 (0.6)	166,558 (91.4)	762 (0.5)	76,581 (92.2)	268 (0.3)
Yes (< 2500 g)	4698 (9.0)	351 (7.5)^a^	15,694 (8.6)	1094 (7.0)^a^	6485 (7.8)	366 (5.6)^a^
	**52,123 (100)**	**613 (1.2)**	**182,252 (100)**	**1856 (1.0)**	**83,066 (100)**	**634 (0.8)**
**Congenital Anomaly**						
Yes	790 (1.5)	153 (19.4)^a^	2857 (1.6)	469 (16.4)^a^	1062 (1.3)	168 (15.8)^a^
No	50,958 (98.5)	450 (0.9)	178,634 (98.4)	1362 (0.8)	81,668 (98.7)	456 (0.6)
	**51,748 (100)**	**603 (1.2)**	**181,491 (100)**	**1831 (1.0)**	**82,730 (100)**	**624 (0.8)**
**Type of Hospital**						
Public	25,051 (48.3)	317 (1.3)^a^	93,969 (51.7)	1135 (1.2)^a^	28,492 (34.4)	320 (1.1)^a^
Mixed	20,679 (39.9)	247 (1.2)	51,443 (28.3)	544 (1.1)	13,217 (16.0)	154 (1.2)^a^
Private	6106 (11.8)	34 (0.6)	36,204 (19.9)	125 (0.3)	41,108 (49.6)	126 (0.3)
	**51,836 (100)**	**598 (1.2)**	**181,616 (100)**	**1804 (1.0)**	**82,817 (100)**	**600 (0.7)**

^a^Statistically significant association by the residue test adjusted to 5% significance. *MHDI* Municipal Human Development Index. *n* Number of participants. %: percentage

The majority of mothers were between 18 and 34 years old, 64.2% (*n* = 203,417) of them were single, legally separated or widowed and the minority, 44.8% (*n* = 142,143) were primiparas which proved to be a protective factor for infant mortality. Regarding prenatal care, 32% (*n* = 101,322) had less than seven consultations, which proved to be a risk factor for infant death in the first year of life. Vaginal delivery occurred in 53.6% (*n* = 170,220) of births and most of them in mixed or private hospitals (53.4%). Newborns with a gestational age of less than 37 weeks, an Apgar Index of less than 7 at the fifth minute of life, low weight and congenital anomaly were also considered risk factors for death (*p* < 0.001). With the exception of the age of 35 or more and type of delivery, all other variables presented significant association with death in the preliminary crude analysis (Table 2), but in the consecutive multivariate analysis, maternal age and previous number of dead children were not associated with the infant mortality outcome.

**Table 2:** Distribution of absolute frequency and association of socio-demographic, perinatal and neonatal characteristics on Infant Mortality in Porto Alegre (Rio Grande do Sul, Brazil) from 2000 to 2017

	TOTAL n(%)	DEATH n(%)	RR [CI95%]	Value of ***p***
**Maternal Age**				
10 to 17 years	24,882 (7.8)	370 (1.5)	1.59[1.42–1.77]	**< 0.001**
≥ 35 years	52,952 (16.7)	493 (0.9)	0.99 [0.90–1.09]	**0.926**
18 to 34 years	239,706 (75.5)	2242 (0.9)	1	
**Marital Status**				
Single/Separated/Widowed	203,417 (64.2)	2317 (1.1)	1.69 [1.56–1.83]	**< 0.001**
Married/stable union	113,361 (35.8)	764 (0.7)	1	
**Parity**				
Primipara	142,143 (44.8)	1180 (0.8)	0.76 [0.71–0.82]	**< 0.001**
Multipara	174,796 (55.2)	1907 (1.1)	1	
**Number of dead children**				
≥ 1	30,460 (9.6)	377 (1.2)	1.30 [1.17–1.45]	**< 0.001**
None	287,004 (90.4)	2727 (1.0)	1	
**Number of Prenatal Care Consultations**				
None	9713 (3.1)	457 (4.7)	9.45 [8.48–10.53]	**< 0.001**
From 1 to 3	25,711 (8.1)	668 (2.6)	5.22 [4.74–5.74]	**< 0.001**
From 4 to 6	65,898 (20.8)	874 (1.3)	2.66 [2.44–2.91]	**< 0.001**
7 and more	215,170 (68.0)	1071 (0.5)	1	
**Birth**				
Vaginal	170,220 (53.6)	1671 (1.0)	1.0 [0.94–1.08]	**0.828**
Cesarean	147,320 (46.4)	1435 (1.0)	1	
**Gestational Age**				
Less than 27 weeks	1317 (0.4)	749 (56.9)	124.96 [116.31–134.25]	**< 0.001**
28 to 31 weeks	2631 (0.8)	397 (15.1)	33.15 [29.83–36.85]	**< 0.001**
32 to 36 weeks	26,244 (8.3)	587 (2.2)	4.91 [4.46–5.41]	**< 0.001**
37 weeks or more	286,735 (90.5)	1305 (0.5)	1	
**Apgar at 5 min**				
< 7	3587 (1.1)	940 (26.2)	41.19 [38.39–44.19]	**< 0.001**
≥ 7	312,140 (98.9)	1986 (0.6)	1	
**Low Weight**				
No (≥2500 g)	290,659 (91.5)	1294 (0.4)	0.07 [0.06–0.07]	**< 0.001**
Yes (< 2500 g)	26,886 (8.5)	1813 (6.7)	1	
**Congenital Anomaly**				
Yes	4711 (1.5)	791 (16.8)	23.02 [21.34–24.83]	**< 0.001**
No	311,361 (98.5)	2271 (0.7)	1	
**Newborn Sex**				
Female	154,886 (48.8)	1411 (0.9)	0.88 [0.82–0.95]	**< 0.001**
Male	162,630 (51.2)	1680 (1.0)	1	
**Type of Hospital**				
Public	147,558 (46.6)	1774 (1.2)	3.50 [3.09–3.97]	**< 0.001**
Mixed	85,357 (27.0)	945 (1.1)	3.23 [2.83–3.69]	**< 0.001**
Private	83,456 (26.4)	286 (0.3)	1	

Dependent variable: death. *RR* Relative risk, *CI95%* 95% confidence interval. *n* Number of participants. %: percentage. <: less

In the bivariate analysis, through Poisson’s simple regression, the maternal education equal or superior to 12 years of study was a protective factor for death. In relation to the general MHDI in its three components, the lower its classification, the higher was the risk of child death in the first year of life. Children of mothers residing in the medium MHDI score died 1.54 times more when compared to those of mothers with very high MHDI scores. On the other hand, children of mothers residing in macro-regions with low MHDIE died 1.66 times more often when compared to those of mothers with very high MHDIE (Table 3).

**Table 3:** Association of the MHDI (and its components) and maternal education on Infant Mortality (unadjusted model) in Porto Alegre (Rio Grande do Sul, Brazil) in the period 2000 to 2017

	Total (n)	Death n(%)	RR [CI95%]	Value of ***p***
**MHDI**				
Medium	52,123	613 (1.2)	1.54 [1.38–1.72]	**< 0.001**
High	182,252	1856 (1.0)	1.33 [1.22–1.46]	**< 0.001**
Very high	83,066	634 (0.8)	1	
**MHDII**				
Medium	31,958	381 (1.2)	1.40 [1.25–1.57]	**< 0.001**
High	137,472	1466 (1.1)	1.26 [1.17–1.35]	**< 0.001**
Very high	148,011	1256 (0.8)	1	
**MHDIL**				
High	11,554	139 (1.2)	1.24 [1.05–1.47]	**0.012**
Very high	305,887	2964 (1.0)	1	
**MHDIE**				
Low	59,957	704 (1.2)	1.66 [1.47–1.87]	**< 0.001**
Medium	136,948	1451 (1.1)	1.49 [1.34–1.66]	**< 0.001**
High	59,988	519 (0.9)	1.22 [1.07–1.39]	**0.002**
Very high	60,548	429 (0.7)	1	
**Maternal Education**				
< 8 years	92,914	1375 (1.5)	2.75 [2.48–3.05]	**< 0.001**
≥ 8 to 11 years	134,210	1219 (0.9)	1.69 [1.52–1.87]	**< 0.001**
	89,143	480 (0.5)	1	

Dependent variable: death. *RR* Relative risk, *CI95%* 95% confidence interval, *MHDI* Municipal Human Development Index, *MHDII* Income component of the Municipal Human Development Index, *MHDIL* Longevity component of the Municipal Human Development Index, *MHDIE* Education component of the Municipal Human Development Index, *n* Number of participants. %: percentage. <: less

The MHDI and its components were analyzed separately with the other variables, so an adjusted model with MHDI and schooling, a model with MHDII and schooling, a model with MHDIL and schooling and finally a model with MHDIE and schooling through multiple Poisson regression adjusting for statistically significant variables in the bivariate analysis to assess the influence of MHDI and maternal education on infant mortality. The first column of Table 4 describes the classifications of each MHDI, as well as maternal education. The MHDI with a medium classification and its MHDIE component with a low classification remained associated with infant death in the first year of life (*p* = 0.036 and *p* = 0.037 respectively), both presenting a 16% higher risk of death. The analysis of maternal education showed that a number less than 8 years of study maintained an association with infant death (*p* < 0.001) both in the model with the general MHDI and in its 3 components, presenting a 37 to 40% higher risk of death (Table 4).

**Table 4:** Association of MHDI, MHDII, MHDIL, MHDIE and its classification (in four different robust Poisson regression models) and maternal education on Infant Mortality (adjusted models)^a^ in Porto Alegre (Rio Grande do Sul, Brazil) in the period 2000 to 2017

	MHDI		MHDII		MHDIL		MHDIE	
	RR [CI95%]	***p***	RR [CI95%]	***p***	RR [CI95%]	***p***	RR [CI95%]	***p***
**Classification of MHDI**								
Low	–	–	–	–	–	–	1.16 [1.01–1.34]	**0.037**
Medium	1.16 [1.01–1.33]	**0.036**	1.09 [0.96–1.24]	0.170	–	–	1.07 [0.94–1.22]	0.305
High	1.03 [0.91–1.15]	0.637	1.04 [0.95–1.14]	0.360	0.99 [0.83–1.20]	0.978	0.92 [0.78–1.07]	0.284
Very high	1		1		1		1	
**Maternal Education**								
< 8 years	1.38 [1.20–1.59]	**< 0.001**	1.39 [1.20–1.60]	**< 0.001**	1.40 [1.22–1.61]	**< 0.001**	1.37 [1.19–1.58]	**< 0.001**
≥ 8 to 11 years	1.04 [0.91–1.19]	0.532	1.05 [0.92–1.19]	0.482	1.06 [0.93–1.20]	0.394	1.04 [0.91–1.18]	0.565
≥ 12 years	1		1		1		1	

Dependent variable: death. *RR* Relative risk, *CI95%* 95% confidence interval, *MHDI* Municipal Human Development Index, *MHDII* Income component of the Municipal Human Development Index, *MHDIL* Longevity component of the Municipal Human Development Index, *MHDIE* Education component of the Municipal Human Development Index, *n* Number of participants. %: percentage. <: less

^a^The model was adjusted for maternal age, parity, number of prenatal care consultations, gestational age, number of dead children, maternal marital status, type of delivery, Apgar, weight (appropriate; low), congenital anomaly, sex of the infant, type of hospital

## Discussion

In recent years, different authors have investigated factors related to infant mortality and maternal-infant health conditions. Regarding the influence of socioeconomic factors, the literature has shown that there are differences according to the place studied [17–19]. In the present study, besides macro-regions with medium MHDI being associated with death (*p* = 0,036), maternal education, individually and jointly analyzed with the MHDI, showed association with the outcome of infant death in the first year of life, particularly for children of mothers with lower maternal education (*p* < 0.001). In relation to other related factors, low maternal age; number of Prenatal Care Consultations; gestational age, weight, gender, congenital anomaly, and Apgar Index (5th minute) of the newborn showed association with IM (p < 0.001) in the municipality of Porto Alegre from 2000 to 2017. These results corroborate previous studies that identified biological and prenatal care factors related to infant death [1, 20–22].

Although most mothers were between 18 and 34 years of age, the highest proportion of child deaths in the first year of life (1.5%) occurred among the children of those under 18. This age group presents a higher risk for complications, since younger women are still in the phase of physical and psychological development and, part of them, performing their studies in primary, secondary or even higher education [23, 24]. Single, legally separated or widowed women presented a higher risk of death than those married or in a stable union (RR = 1.69; CI95% 1.56–1.83). In literature, other studies have described that the condition of women without a fixed partner negatively affects the health of the mother and child, because it generates some degree of suffering for the woman, who may develop, among other conditions, depression, thus influencing the mother-baby interaction. Moreover, it represented a decrease in emotional and economic support for the family [25–27].

As for the type of birth, most women have had vaginal birth. However, the present study did not evaluate the temporal evolution of this variable, since it is known that this percentage has varied according to the year. Although the evidence does not show an association between cesarean section and a decrease in IM, the number of cesarean sections has been steadily increasing in both developing and developed countries [28, 29]. In 2015, for the first time in Brazil, the number of operative deliveries stabilized [6]. The proportion of cesarean sections was higher in macro-regions with better MHDI scores. This ratio, according to different authors, could be explained by the preference for cesarean sections with higher socioeconomic level, white ethnicity and higher maternal education [30, 31]. Moreover, the most visible discrepancies are observed when comparing cesarean rates in public and private hospitals in several regions of Brazil [31, 32]. These rates in the private sector are significantly higher than in the public health system, exceeding 90% in some hospitals [33, 34].

The MHDI has proven to be an indicator that has a relationship with the variables studied. The lower the classification of the MHDI of the macro-region of maternal residence, the worse were the socioeconomic and demographic conditions. Previous studies that evaluated infant mortality in different regions or ethnic groups found similar results [18, 19]. Cruz and collaborators [17] observed that, in Brazil, women living in more developed regions have lower chances of having an early pregnancy. Approximately, an analysis conducted in Nigeria with population data found that lower maternal age, as well as lower education, especially of mothers who had deceased children showed association with child death [18]. In this context, this same association is found with the worst maternal conditions observed in less developed or developing countries, as well as in regions with lower income and limited access to health services [3, 35].

In the same perspective, although studies show that prenatal care coverage in Brazil has increased by up to 98.7%, the percentage of women with less than 7 prenatal visits is frequent and may vary according to the region analyzed [36, 37]. Data regarding the city in this study showed an improvement of this indicator by 41.71% in the period from 2001 to 2017. However, it is known that this percentage varied according to interurban and health care differences, and there may be regions with adequate Prenatal Care Consultations above 80% and others below 65% [38]. Although the present study did not specifically investigate the annual temporal evolution of prenatal care coverage, these percentages are similar to the results found for this variable.

Research that assessed the influence of the HDI of the maternal home region, when at birth, on health outcomes, showed that this indicator when low is associated with worse outcomes with respect to child neuropsychomotor development [39, 40] and also with higher infant mortality rates [19, 41]. In a study with data from 188 countries, Ruiz and collaborators [41] found that IM was correlated with low HDI and low inequality-adjusted HDI, with the latter being more strongly correlated than the former (*Z* = 2.524, *p* = 0.012).

Although higher maternal education does not guarantee protection for infant death in the first year of life, as shown in the analysis, schooling less than 8 years of study represents a risk factor increasing the chance of death by 37 to 40%, both in the MHDI and its three components. That is, even though the higher maternal education level did not show statistical significance in reducing infant mortality in the present study, the low educational level showed an association with infant death. It is assumed that these results are attributed to the worst socioeconomic conditions of that mother and the social and community environment in which she lives with limited access to various resources and health services as well as worse conditions of basic sanitation [22, 42, 43]. Viellas and collaborators [36] found that in Brazil, women with lower education had less prenatal care coverage, fewer prenatal visits and higher utilization of public services. In contrast, they found that those with higher education used more specialized prenatal services, mostly in private services, with monitoring by the same professional throughout pregnancy. In this sense, prenatal care is a process of health education for women and their families, collaborating in the care of pregnancy and children, including, often, the beginning of health care for some individuals, especially adolescents [44].

However, other studies have shown that, in addition to socioeconomic conditions, higher maternal education has improved the child’s health conditions and prevented infant mortality. The higher educational level of mothers, together with their better intellectual capacities, help in health choices, increase the understanding and use of information and improve the perception of health problems [45]. They also invest in more health-beneficial behaviors, both at the individual and child levels, showing better adherence to health recommendations in terms of nutrition, exercise and reduction of harmful habits [46–49].

Some particular features of the study are worth highlighting. The study was a pioneer in the country in analyzing the relationship of the MHDI of the different regions of a municipality with infant mortality. It was also unprecedented in performing a parallel analysis of the influence of maternal schooling and MHDI on IM. Furthermore, it used two well consolidated health information systems in the municipality over an extended period of time (2000–2016), which provided a robust database with higher quality in its processing and analysis. This study was important because it revealed that maternal education was shown to be superior to the MHDI as a predictor of infant mortality, reinforcing that when schooling is very low, it can contribute to risky behaviors, and perhaps in addition to being a social indicator, it should be considered a health indicator for the child, as it helps in the health choices of the child’s mother and adherence to recommendations. With a health indicator, the mother’s educational level could guide the elaboration of public policies, assisting in the screening of risk groups not only by the region of residence, but also by the individual maternal conditions and thus enabling more agile and targeted actions for these individuals of greater risk.

On the other hand, the study presented some limitations. Among them, the non-use of the race/skin color variable, as it was incomplete in some of the years analyzed, and the use of secondary data with pre-established variables and lack of information on serious diseases and maternal characteristics (income, maternal weight and smoking) and more specific neonatal characteristics (breastfeeding). One hypothesis to explain that MHDI did not present a higher risk of death, as well as what has already been evidenced in the literature is that just like the HDI, the MHDI when analyzed in macro-regions can hide areas of low development and social vulnerability. This could have influenced the analyses since the MHDI values are distributed to the macro-region, not including in the analytical process the micro-regions classified with low or very low MHDI. Porto Alegre currently has 335 micro-regions [15], of which many have very low MHDI values, especially when analyzing the education component. However, the database used did not have a list with the address codes for each micro-region.

## Conclusions

The HDI is considered a good predictor of infant mortality by some authors and the analyzes of the present study also confirm an association of the medium MHDI and its low MHDIE component with infant mortality. In addition, it was maternal education with less than 8 years of study that that demonstrated a higher risk of death, revealing itself to be a social determinant with a relevant impact on infant mortality. Thus, it is possible to conclude that maternal education is available information, and it is superior to the MHDI to assess the infant mortality outcome.

## Supplementary Information

**Additional file 1: Figure 1.** Map of the studied city by districts (Porto Alegre, Rio Grande do Sul, Brazil). Map of the studied city by districts (Porto Alegre, Rio Grande do Sul, Brazil). OBSERVAPOA and PROCEMPA, 2016. Public domain. http://observapoa.com.br/default.php?reg=259&p_secao=46

**Additional file 2: Figure 2.** Map of the studied city by Participatory budgeting Regions - macro-regions (Porto Alegre, Rio Grande do Sul, Brazil). Map of the studied city by Participatory budgeting Regions - macro-regions (Porto Alegre, Rio Grande do Sul, Brazil). OBSERVAPOA and PROCEMPA, 2016. Public domain. http://lproweb.procempa.com.br/pmpa/prefpoa/observatorio/usu_doc/sem_grids.pdf

**Additional file 3: Table 1.** Planning Management Regions and Participatory budgeting Regions (macro-regions) and their corresponding MHDI values and their 3 components of Porto Alegre (Rio Grande do Sul, Brazil). Planning Management Regions and Participatory budgeting Regions (macro-regions) and their corresponding MHDI values and their 3 components of Porto Alegre (Rio Grande do Sul, Brazil). OBSERVAPOA and PROCEMPA, 2016. http://observapoa.com.br/default.php?reg=259&p_secao=46

**Additional file 4: Graphic 1.** Infant Mortality Rate per thousand live births according to the Municipal Human Development Index (MHDI) classification in Porto Alegre (Rio Grande do Sul, Brazil) from 2000 to 2017. Infant Mortality Rate per thousand live births according to the Municipal Human Development Index (MHDI) classification in Porto Alegre (Rio Grande do Sul, Brazil) from 2000 to 2017. Graphic made by the author.

**Additional file 5.** Title of data: Study dataset. Dataset generated by the similarity of the two databases related to the current study.

## Data Availability

The dataset generated by the likage of the two databases related to the current study are available in the “Additional file 5”. For the purpose of maintaining the confidentiality of individuals, the variables number of the Declaration of Live Birth, mother’s name and home address number were excluded.

## Abbreviations

IM: Infant mortality
MDG 4: Millennium Development Goal 4
MDG 3: Millennium Development Goal 3
HDI: Human Development Index
MHDI: Municipal Human Development Index
MHDII: Income Component of the Municipal Human Development Index
MHDIL: Longevity Component of the Municipal Human Development Index
MHDIE: Education Component of the Municipal Human Development Index
SINASC: Live Births Information System
SIM: Mortality Information System
IBGE: Brazilian Institute of Geography and Statistics
