# Supplementary material for: The influence of the municipal human development index and maternal education on infant mortality: an investigation in a retrospective cohort study in the extreme south of Brazil
Source: BMC Public Health. 2021 Jan 22;21:194. doi: 10.1186/s12889-021-10226-9 (PMC7821400; doi:10.1186/s12889-021-10226-9)
Supplement: Supplementary file 1 — Additional file 1: Figure 1. Map of the studied city by districts (Porto Alegre, Rio Grande do Sul, Brazil). Map of the studied city by districts (Porto Alegre, Rio Grande do Sul, Brazil). OBSERVAPOA and PROCEMPA, 2016. Public domain. http://observapoa.com.br/default.php?reg=259&p_secao=46 [file 12889_2021_10226_MOESM1_ESM.docx]

**Additional File 1**

Figure 1: Map of the studied city by districts (Porto Alegre, Rio Grande do Sul, Brazil).


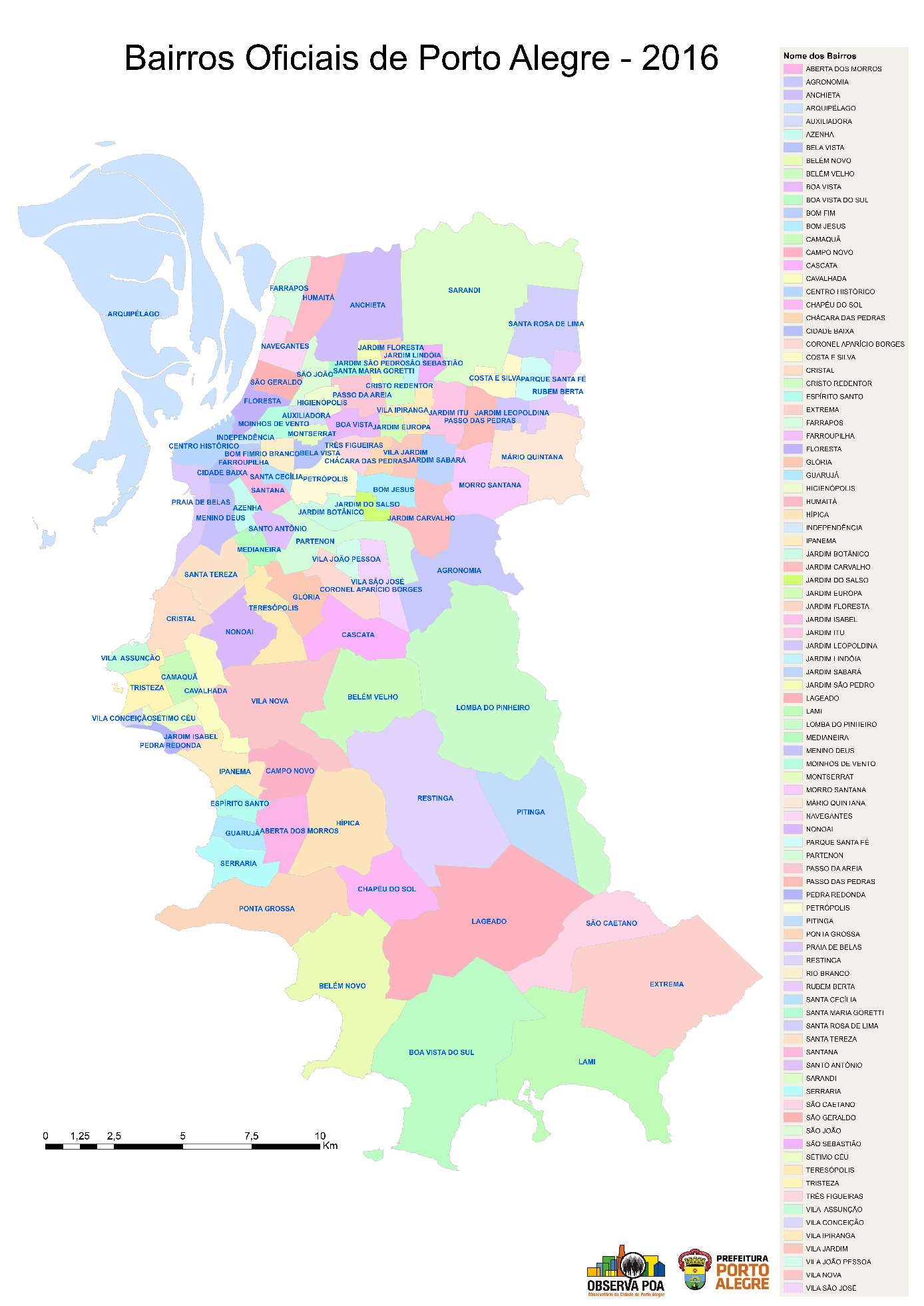


**Name of Districts**

**Official Districts of Porto Alegre - 2016**

*Source: OBSERVAPOA and PROCEMPA, 2016. Public domain.*

*http://observapoa.com.br/default.php?reg=259&p_secao=46*
