# Supplementary material for: The influence of the municipal human development index and maternal education on infant mortality: an investigation in a retrospective cohort study in the extreme south of Brazil
Source: BMC Public Health. 2021 Jan 22;21:194. doi: 10.1186/s12889-021-10226-9 (PMC7821400; doi:10.1186/s12889-021-10226-9)
Supplement: Supplementary file 2 — Additional file 2: Figure 2. Map of the studied city by Participatory budgeting Regions - macro-regions (Porto Alegre, Rio Grande do Sul, Brazil). Map of the studied city by Participatory budgeting Regions - macro-regions (Porto Alegre, Rio Grande do Sul, Brazil). OBSERVAPOA and PROCEMPA, 2016. Public domain. http://lproweb.procempa.com.br/pmpa/prefpoa/observatorio/usu_doc/sem_grids.pdf [file 12889_2021_10226_MOESM2_ESM.docx]

**Additional File 2**

Figure 2: Map of the studied city by Participatory budgeting Regions - macro-regions (Porto Alegre, Rio Grande do Sul, Brazil).


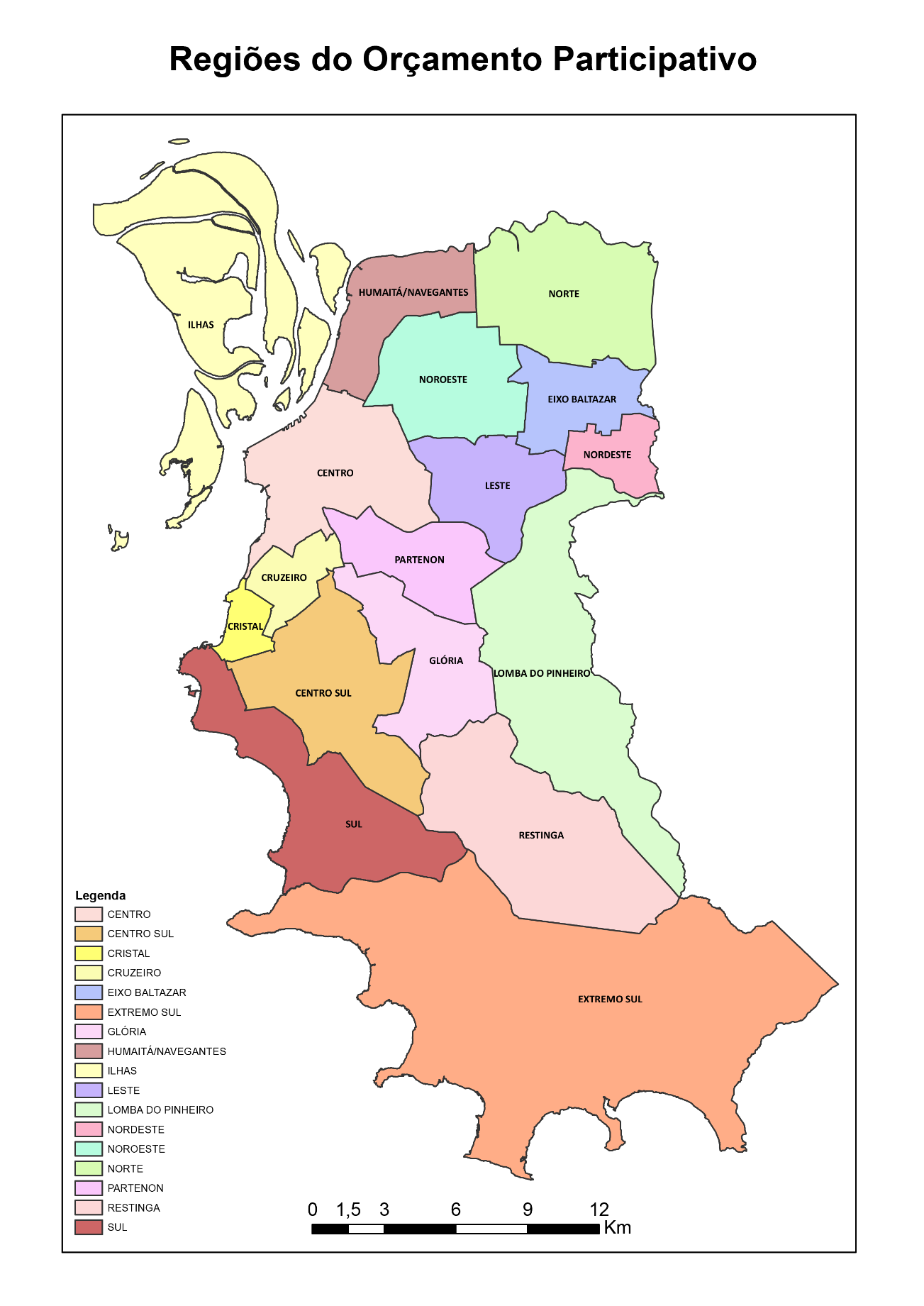


**Participatory Budgeting Regions**

*Source: OBSERVAPOA and PROCEMPA, 2016.* *Public domain.*

*http://lproweb.procempa.com.br/pmpa/prefpoa/observatorio/usu_doc/sem_grids.pdf*
