# Supplementary material for: The influence of the municipal human development index and maternal education on infant mortality: an investigation in a retrospective cohort study in the extreme south of Brazil
Source: BMC Public Health. 2021 Jan 22;21:194. doi: 10.1186/s12889-021-10226-9 (PMC7821400; doi:10.1186/s12889-021-10226-9)
Supplement: Supplementary file 3 — Additional file 3: Table 1. Planning Management Regions and Participatory budgeting Regions (macro-regions) and their corresponding MHDI values and their 3 components of Porto Alegre (Rio Grande do Sul, Brazil). Planning Management Regions and Participatory budgeting Regions (macro-regions) and their corresponding MHDI values and their 3 components of Porto Alegre (Rio Grande do Sul, Brazil). OBSERVAPOA and PROCEMPA, 2016. http://observapoa.com.br/default.php?reg=259&p_secao=46 [file 12889_2021_10226_MOESM3_ESM.docx]

**Additional File 3**

Table 1. **Planning Management Regions** and Participatory budgeting Regions (macro-regions) and their corresponding MHDI values and their 3 components of Porto Alegre (Rio Grande do Sul, Brazil).

| Planning Management Regions | MACRO-REGION | MHDI | MHDII | MHDIL  2010 | MHDIE |
| --- | --- | --- | --- | --- | --- |
|  |  | 2010 | 2010 |  | 2010 |
|  |  |  |  |  |  |
| Center | Center | 0.935 | 0.984 | 0.929 | 0.894 |
| Humaitá/ Navegantes/ Islands and Northwest | Humaitá / Navegantes | 0.765 | 0.807 | 0.854 | 0.649 |
|  | Northwest | 0.89 | 0.921 | 0.913 | 0.837 |
|  | Islands | 0.659 | 0.705 | 0.805 | 0.504 |
| North and eixo Baltazar | North | 0.729 | 0.750 | 0.833 | 0.620 |
|  | Eixo Baltazar | 0.779 | 0.785 | 0.849 | 0.709 |
| East/Northeast | East | 0.777 | 0.831 | 0.874 | 0.646 |
|  | Northeast | 0.638 | 0.655 | 0.778 | 0.509 |
| Glória/ Cruzeiro and Cristal | Cristal | 0.809 | 0.858 | 0.887 | 0.695 |
|  | Cruzeiro | 0.747 | 0.797 | 0.851 | 0.614 |
|  | Glória | 0.733 | 0.775 | 0.841 | 0.605 |
| Center-South and South | Center-South | 0.797 | 0.817 | 0.868 | 0.713 |
|  | South | 0.843 | 0.897 | 0.892 | 0.750 |
| Lomba do Pinheiro/ Partenon | Lomba do Pinheiro | 0.683 | 0.691 | 0.806 | 0.571 |
|  | Partenon | 0.764 | 0.795 | 0.851 | 0.659 |
| Restinga/ Far South | Restinga | 0.685 | 0.705 | 0.803 | 0.567 |
|  | Far South | 0.714 | 0.756 | 0.835 | 0.576 |

*RS: Rio Grande do Sul. MHDI: Municipal Human Development Index; MHDII: Income component of the Municipal Human Development Index; MHDIL: Longevity component of the Municipal Human Development Index; MHDIE: Education component of the Municipal Human Development Index.*

*Source: Adapted from OBSERVAPOA and PROCEMPA, 2016.*

*http://observapoa.com.br/default.php?reg=259&p_secao=46*
