# Supplementary material for: The influence of the municipal human development index and maternal education on infant mortality: an investigation in a retrospective cohort study in the extreme south of Brazil
Source: BMC Public Health. 2021 Jan 22;21:194. doi: 10.1186/s12889-021-10226-9 (PMC7821400; doi:10.1186/s12889-021-10226-9)
Supplement: Supplementary file 4 — Additional file 4: Graphic 1. Infant Mortality Rate per thousand live births according to the Municipal Human Development Index (MHDI) classification in Porto Alegre (Rio Grande do Sul, Brazil) from 2000 to 2017. Infant Mortality Rate per thousand live births according to the Municipal Human Development Index (MHDI) classification in Porto Alegre (Rio Grande do Sul, Brazil) from 2000 to 2017. Graphic made by the author. [file 12889_2021_10226_MOESM4_ESM.docx]

**Additional File 4**

Graphic 1. Infant Mortality Rate per thousand live births according to the Municipal Human Development Index (MHDI) classification in Porto Alegre (Rio Grande do Sul, Brazil) from 2000 to 2017.

*MHDI: Municipal Human Development Index.*

*The MHDI is an index used in Brazil that allows to know the reality of human development in the Brazilian territory.* *It is previously calculated and established by the United Nations Development Program Brazil (UNDP Brazil), the Institute for Applied Economic Research (IPEA) and the João Pinheiro Foundation*

**Medium: IDHM 0.6-0.699; High: IDHM 0.7-0.799; Very high: IDHM 0.8-1.0.*

*Source: Graphic made by the author.*
